# A reconfigurable arbitrary waveform generator using PWM modulation for ultrasound research

**DOI:** 10.1186/1475-925X-12-24

**Published:** 2013-03-20

**Authors:** Amauri A Assef, Joaquim M Maia, Fábio K Schneider, Vera LSN Button, Eduardo T Costa

**Affiliations:** 1 Electrical/Electronic Engineering Department and the Graduate School of Electrical Engineering and Applied Computer Sciences (DAELT – DAELN – CPGEI), Federal University of Technology – Paraná (UTFPR), Curitiba, PR, Brazil; 2 Biomedical Engineering Department of the School of Electrical and Computer Engineering (DEB/FEEC) and Biomedical Engineering Centre (CEB), University of Campinas (UNICAMP), Campinas, SP, Brazil

**Keywords:** Ultrasound, FPGA, Arbitrary waveform generator, Transmit beamformer

## Abstract

**Background:**

In ultrasound imaging systems, the digital transmit beamformer is a critical module that generates accurate control over several transmission parameters. However, such transmit front-end module is not typically accessible to ultrasound researchers. To overcome this difficulty, we have been developing a compact and fully programmable digital transmit system using the pulse-width modulation (PWM) technique for generating simultaneous arbitrary waveforms, specifically designed for research purposes.

**Methods:**

In this paper we present a reconfigurable arbitrary waveform generator (RAWG) for ultrasound research applications that exploits a high frequency PWM scheme implemented in a low-cost FPGA, taking advantage of its flexibility and parallel processing capability for independent controlling of multiple transmission parameters. The 8-channel platform consists of a FPGA-based development board including an USB 2.0 interface and an arbitrary waveform generator board with eight MD2130 beamformer source drivers for individual control of waveform, amplitude apodization, phase angle and time delay trigger.

**Results:**

To evaluate the efficiency of our system, we used equivalent RC loads (1 kΩ and 220 pF) to produce arbitrary excitation waveforms with the Gaussian and Tukey profiles. The PWM carrier frequency was set at 160 MHz featuring high resolution while keeping a minimum time delay of 3.125 ns between pulses to enable the acoustic beam to be focused and/or steered electronically. Preliminary experimental results show that the RAWG can produce complex arbitrary pulses with amplitude over 100 Vpp and central frequency up to 20 MHz with satisfactory linearity of the amplitude apodization, as well as focusing phase adjustment capability with angular resolution of 7.5°.

**Conclusions:**

The initial results of this study showed that the proposed research system is suitable for generating simultaneous arbitrary waveforms, providing extensive user control with direct digital access to the various transmission parameters needed to explore alternative ultrasound transmission techniques.

## Background

In medical ultrasound (US) imaging systems, also called scanners, the transmit (TX) beamformer represents an important segment that generates high-voltage (HV) pulsed signals to effectively excite the transducer for a satisfactory signal-to-noise ratio (SNR) [1,2]. Although commercial US systems have been typically used by research laboratories for the development and experimental test of new investigation methods for transmission of US, these systems do not always fit the needs for testing the proposed novel approaches [3]. With limited programmability and flexibility, research users of these machines who may wish to evaluate alternative transmission techniques cannot have access to various US transmission parameters during pulse-echo experiments, because their typical architecture is often “closed” and available only for system engineers [4,5].

**Figure 1:**
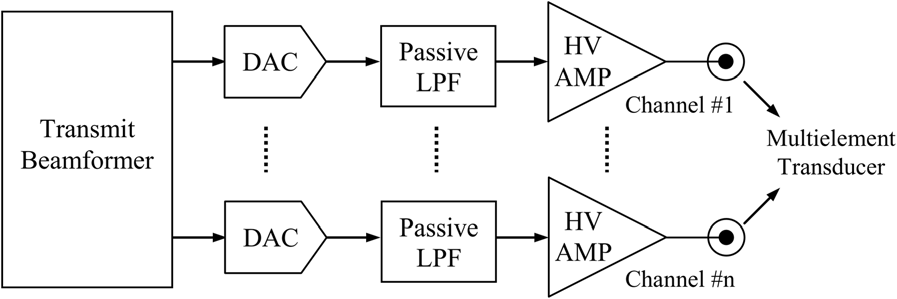
Block diagram of a transmit beamformer with multichannel arbitrary waveform generator.

The hardware strategy to excite an US transducer element with high voltage swings as large as 200 Vpp and with peak currents up to 2 A [6] is a critical consideration in the transmitter design which involves a trade-off between electronics complexity and system performance to optimize the image quality for each US application [7-10]. In modern US systems the advanced excitation scheme employs arbitrary waveform generators (AWGs) [10], typically controlled by analog and digital custom application-specific integrated circuits (ASICs) or, more recently, reconfigurable technologies based on field-programmable gate arrays (FPGAs) [11]. Independent excitation of each piezoelectric element in a multielement US transducer can be performed with low second order harmonic distortions for modulated excitation imaging [10]. However, as illustrated in Figure 1, this transmit technique requires additional expensive electronics, e.g., digital-to-analog converters (DACs), low-pass filtering (LPF) and linear high-voltage amplifiers (HV AMP) to translate the digital waveform to an amplified analog signal to drive the transducer elements, and thus, generally reserved for more expensive and less portable high performance US systems. As a result, most of these systems do not use this transmit beamformer technique, but instead use unipolar, bipolar or multilevel high-voltage pulsers to generate the necessary transmit signals [6].

In recent years, some commercial US machines have been introduced with different implementation to enable researchers direct control of multielement probes [5,12]. A significant example is represented by the Verasonics research scanner (Verasonics Inc., WA, USA) that is built on an open-architecture software platform that can be configured to operate in various modes required for research, such as unfocused broad beam emissions, which can be used to increase the frame rate over conventional focused beam approaches [13]. Another commercial US equipment designed for medical and industrial applications is the OPEN System (Lecoeur Electronique Corp. Chuelles, France), based on a modular architecture with multiple dedicated electronics boards that includes programmable analog transmitters and an USB 2.0 interface to a host computer.

On the other hand, only few US platforms have been specifically developed for research purposes and a need exists for open architectures for direct access to the transmission parameters with an independent excitation scheme for each channel [14-20]. One of these is the ULtrasound Advanced Open Platform (ULA-OP) [14], which applies the sigma-delta technique combined with the high-speed of the low-voltage differential signaling (LVDS) channels integrated on FPGAs to synthesize arbitrary waveforms with output amplitude operating up to 24 Vpp. Alternatively, another method for generating arbitrary waveforms is presented in paper [15]. Here, Jensen *et al.* described the Remotely Accessible Software configurable Multichannel Ultrasound Sampling (RASMUS) system, a high-level US research scanner for real-time synthetic aperture acquisition data capable of different arbitrary emission strategies, where the individual synthesized waveforms are stored in a 128-ksample pulse RAM, controlled by two FPGAs, and connected to a 40 MHz, 12-bit digital-to-analog converter (DAC).

In this paper we present a reconfigurable arbitrary waveform generator (RAWG) that exploits the pulse-width modulation (PWM) technique implemented in a low-cost FPGA for independent control of multiple transmission parameters. All electronics necessary to control 8-channel simultaneously were integrated in two boards, which can be connected to any PC through the USB 2.0 high speed interface. The novel architecture introduces the possibility of extensive user control over the amplitude apodization and excitation waveform of individual elements in a multielement transducer, as well as the time delays and phase adjustment between them, to enable the acoustic beam to be focused and/or steered electronically.

## Methods

### Reconfigurable Arbitrary Waveform Generator (RAWG)

**Figure 2:**
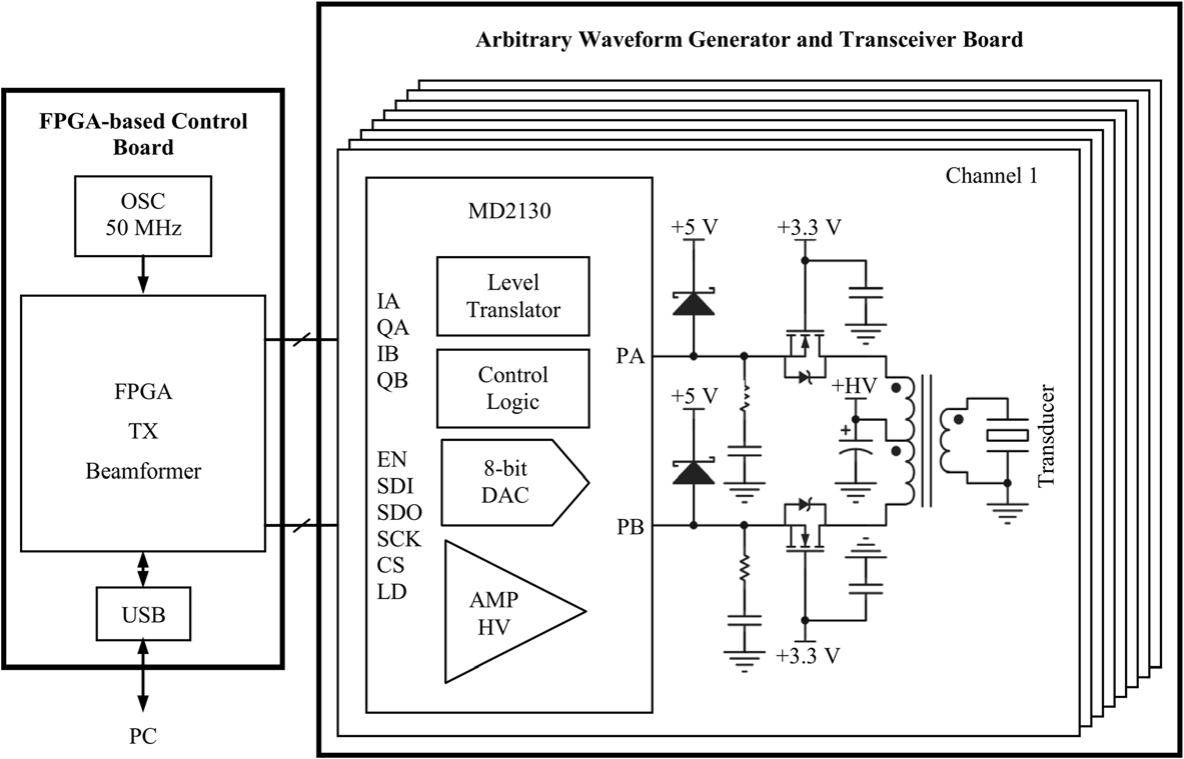
Block diagram of the reconfigurable arbitrary waveform generator.

In Figure 2, the block diagram of the pulse generator is illustrated. The reconfigurable arbitrary waveform generator (RAWG) consists of a personal computer (PC) for configuration through an USB 2.0 interface and two printed circuit boards (PCB): a digital FPGA-based control board and an AWG and analog transceiver board [21].

The digital transmit and control board (Cyclone III FPGA Development Board, Altera, CA, USA) uses an Altera EP3C120 FPGA that works at 320 MHz as the central processor. The FPGA has 531 user I/O pins, 119,088 logic elements and a total of 3,981,312 bits of internal RAM, which is crucial for handling a large amount of synthesized arbitrary waveforms data [19].

The AWG board includes eight high-speed arbitrary waveform push-pull source driver MD2130 (Supertex Inc., CA, USA), high-voltage MOSFETs, US pulse transformers for impedance matching and T/R switches for interface with commercial analog front-end (AFE) evaluation modules, as described by Assef *et al.*[21]. The communication between the FPGA and the US beamforming source drivers is performed by eight high-speed serial peripheral interface (SPI) to achieve fast updating, through a 172-pin High-Speed Mezzanine Card (HSMC) connector (Samtec Inc., IN, USA).

The FPGA circuit not only generates accurate timing for each serial data and clock to set and change the TX parameters (amplitude apodization and phase adjustment), but also provides a suitable scheme for the eight high-speed PWM control waveforms. The digital waveforms data, synthesized in two in-phase (IA and IB) and quadrature (QA and QB) PWM signals, can be independently driven to each channel with a fully programmable sequence, including output timing, frequency, cycle in the burst and waveform envelope. A state machine in the FPGA allows easy control to produce the individual excitation waveform that can be transferred from the PC through the USB channel, according to highly flexible transmission strategies using concatenated chain of look-up tables (LUTs). In this case, the FPGA transfers the selected digital arbitrary waveform PWM data to the eight MD2130 integrated circuits (ICs), which convert the PWM signal into a complex high voltage analog waveform.

The essence of focusing an US beam is to align the pressure fields from all parts of the aperture to arrive at the field point at the same time. This can be done by the use of electronics delays for multielement arrays. During ultrasonic transmission, the FPGA triggers the eight MD2130 to excite a group of transducer elements at different times depending on the depth and focal point with time delay resolution of 3.125 ns. For an image plane on the x-z plane at y = 0, the time delay (*τ*_*di*_) to use on each element *i* (*i* = 1, 2, 3, …, *N*) [1] can be obtained by [22]

(1) $$\tau_{\text{di}}=\frac{1}{c}\left(\sqrt{{\left(x_{i}-x_{f}\right)}^{2}+{\left(z_{i}-z_{f}\right)}^{2}}\right)-\sqrt{{\left(x_{c}-x_{f}\right)}^{2}+{\left(z_{i}-z_{f}\right)}^{2}}$$

where (*x*_*c*_ , *z*_*c*_) is the reference center point of the aperture, (*x*_*f*_ , *z*_*f*_) is the point of the focal point, (*x*_*i*_ , *z*_*i*_) is the center for the physical element number *i*, and *c* is the speed of sound.

**Figure 3:**
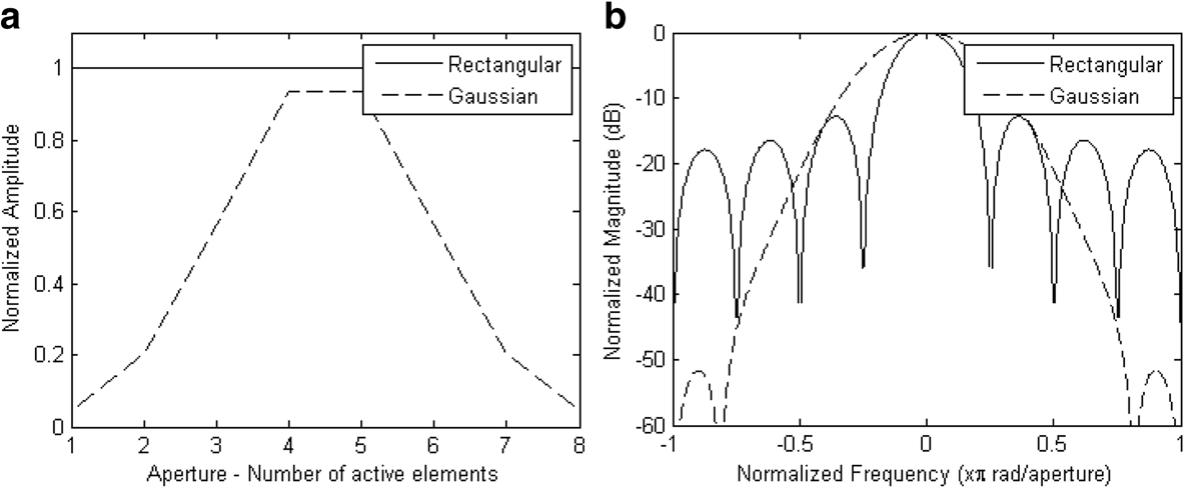
**Normalized responses for the rectangular (solid lines) and the Gaussian (dashed lines) excitation profiles for an active aperture of 8 elements in the array. a**) Normalized transmit apodization coefficients. **b**) Magnitude of the Fourier transform of both window types.

The RAWG also generates the apodization weight *w*_*i*_ for each element *i* for a high signal-to-noise ratio (SNR) [1,23]. The aperture apodization is a well-established technique applied to improve spatial resolution and reduce side lobes artifacts in the radiated beam pattern of the array, as well as a good penetration depth to increase the image quality [1,24]. Typically, a Gaussian shaped function is used for amplitude apodization [22], as can be seen in the example given in Figure 3, where the solid lines represent the rectangular window responses and the dashed lines represent the Gaussian window responses for an active aperture of 8 elements in the array. Figure 3(a) shows the normalized transmit apodization coefficients and Figure 3(b) shows the influence of aperture apodization on the magnitude of the Fourier transform of both window types. Although the main lobe has been widened only by a small amount, as presented in [25], the magnitude of the first side lobe relative to the main lobe is reduced at levels lower than −50 dB.

The reconfiguration of multiple transmission parameters implemented in the FPGA can be easily adjustable for different research approaches through a proprietary software with an user friendly interface [21]. These parameters include excitation waveform, pulse repetition frequency (PRF), phase angle and time delay. The angular resolution of the RAWG is 7.5° per step with total range of 48 steps (360°) and the push-pull output current *I*_*out*_ is given by

(2) $$I_{\text{out}}=\left(\frac{\text{DAC}}{255}\right)I_{\text{max}}+I_{\text{oo}}$$

where *DAC* is the value of the 8-bit MD2130 DAC register, *I*_*max*_ is the full scale output peak current (from 2.7 A to 3.3 A) and *I*_*oo*_ is the output current offset (from 0.5 mA to 1.0 mA) [26]. Thereby, any user change in the beamforming phase angles or apodization amplitudes is updated automatically in the 16-bit data serial register and then transferred simultaneously to the MD2130 devices by the SPI, which works with a serial clock maximum frequency of 20 MHz. Each data serial register includes two most significant bits (MSB) for command options, eight bits for the DAC waveform amplitude control (0 – 255) and the six least significant bits (LSB) for the phase angle adjustment (0 – 48), as presented by Supertex Inc. [26].

### Generation of complex arbitrary waveform

In order to synthesize the transmission waveforms, Eq. (3) and (4) (adapted from [23]) were used to calculate the in phase – *i(n)* and quadrature – *q(n)* signals, respectively, with the Gaussian profile:

(3) $$i\left(n\right)=\text{exp}\left[-{\left(\frac{n-N/2}{B}\right)}^{2}\right]\;\text{cos}\left(2\pi \frac{n}{T}\right),1\;\underset{\leq }{\leq }\;n\;\underset{\leq }{<}\;N$$

(4) $$q\left(n\right)=\text{exp}\left[-{\left(\frac{n-N/2}{B}\right)}^{2}\right]\;\text{sen}\left(2\pi \frac{n}{T}\right),1\;\underset{\leq }{<}\;n\;\underset{\leq }{<}\;N$$

where *N* is the total number of samples (sampling rate/output center frequency), *n* is the sample position, *B* is the Gaussian factor, and *T* is the period of the output waveform.

**Figure 4:**
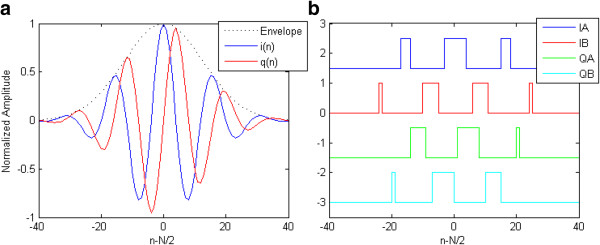
**Simulated waveforms used for generating the high-speed digital PWM excitation signals, considering a sampling rate of 160 MHz to transmit a 20 MHz center frequency waveform.** (**a**) In phase – *i(n)* and quadrature – *q(n)* waveforms. (**b**) Digital PWM signals IA, IB, QA and QB.

For example, Figure 4(a) illustrates the simulated *i(n)* and *q(n)* signals considering a sampling rate of 160 MHz to transmit a 20 MHz center frequency waveform (8 samples per cycle of US output waveform) with 50% relative bandwidth (*B* = 17). Figure 4(b) shows the simulated results of the four digital PWM signals (IA, IB, QA and QB) that should be internally stored in the FPGA LUTs.

## Results

**Figure 5:**
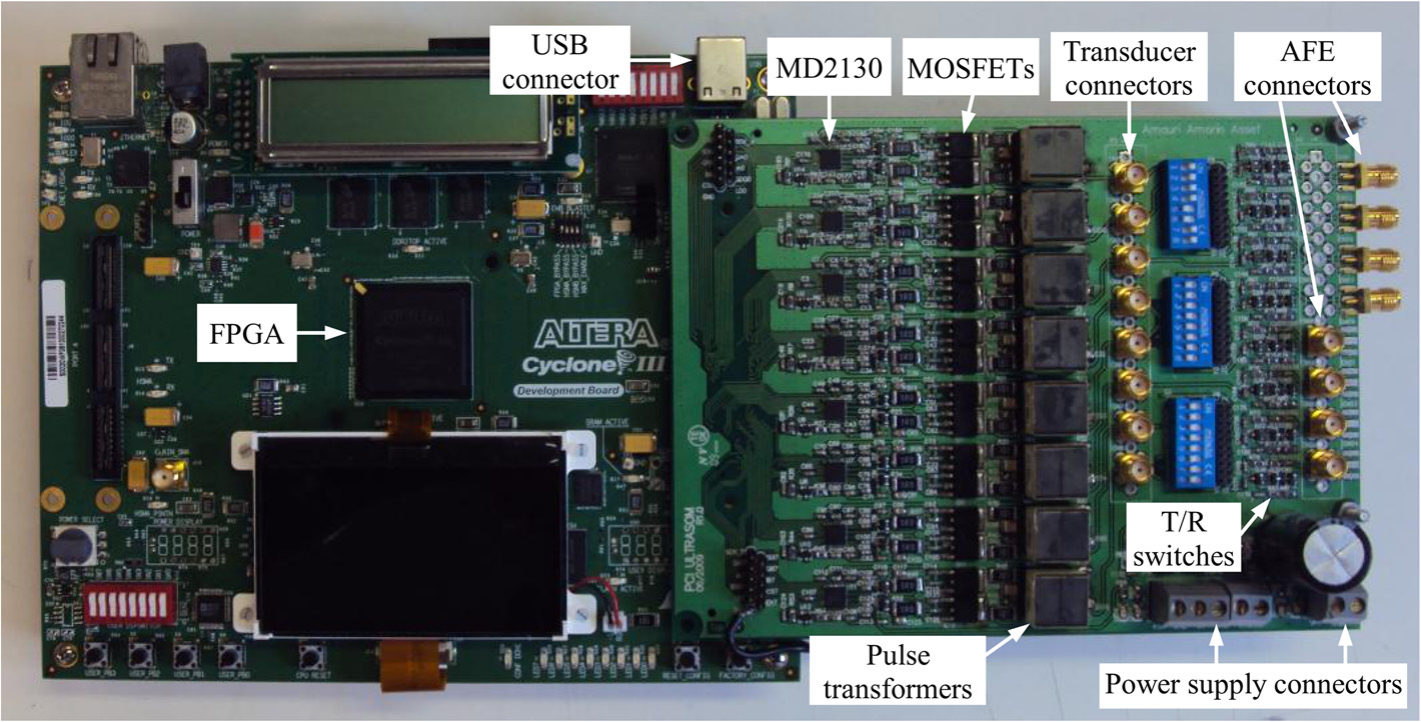
Reconfigurable arbitrary waveform generator architecture showing the main components of the system.

The implemented RAWG hardware architecture is shown in Figure 5 with a detailed labelling of the individual units. It includes the FPGA-based board for command and central control with a high-speed USB 2.0 connector and the AWG board with the MD2130 devices, MOSFETs, transformers, SMA connectors for transducers, T/R switches, output SMA connectors for AFE evaluation modules and power supply connectors. Additional information about the hardware architecture can be found in paper [21].

The performance of the AWG was evaluated using RC loads (1 kΩ and 220 pF) and the system was set to an excitation waveform with the Gaussian profile. The power supply was set to +70 V for high-voltage pulse generation and the PRF was set to 1 kHz. The waveforms shown in this paper were recorded by a digital oscilloscope MSO6034A (Agilent Technologies, CA, USA).

**Figure 6:**
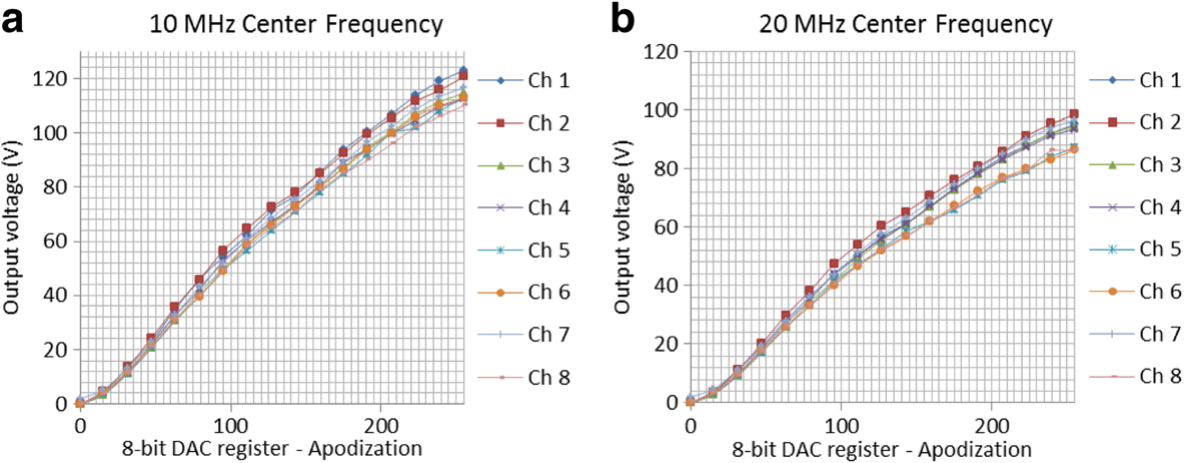
Measurements results from channels 1 to 8 for apodization DAC range value from 0 to 255 with the increment of 15 steps for (a) 10 MHz and (b) 20 MHz center frequency pulses.

The arbitrary pulse generators have been characterized by measuring the peak voltage in channels 1 to 8, applying excitation pulses of −6 dB bandwidth with relative bandwidth of 50%. Figures 6(a) and 6(b) allow analysis of the apodization DAC with satisfactory linearity by plotting the output voltage versus the DAC value register range value from 0 to 255 with the increment of 15 steps for 10 MHz and 20 MHz center frequency pulses, respectively. The maximum output frequency that the RAWG is capable of generating is 20 MHz and a decrease in the output amplitude over the 10 MHz to 20 MHz can be noted.

**Figure 7:**
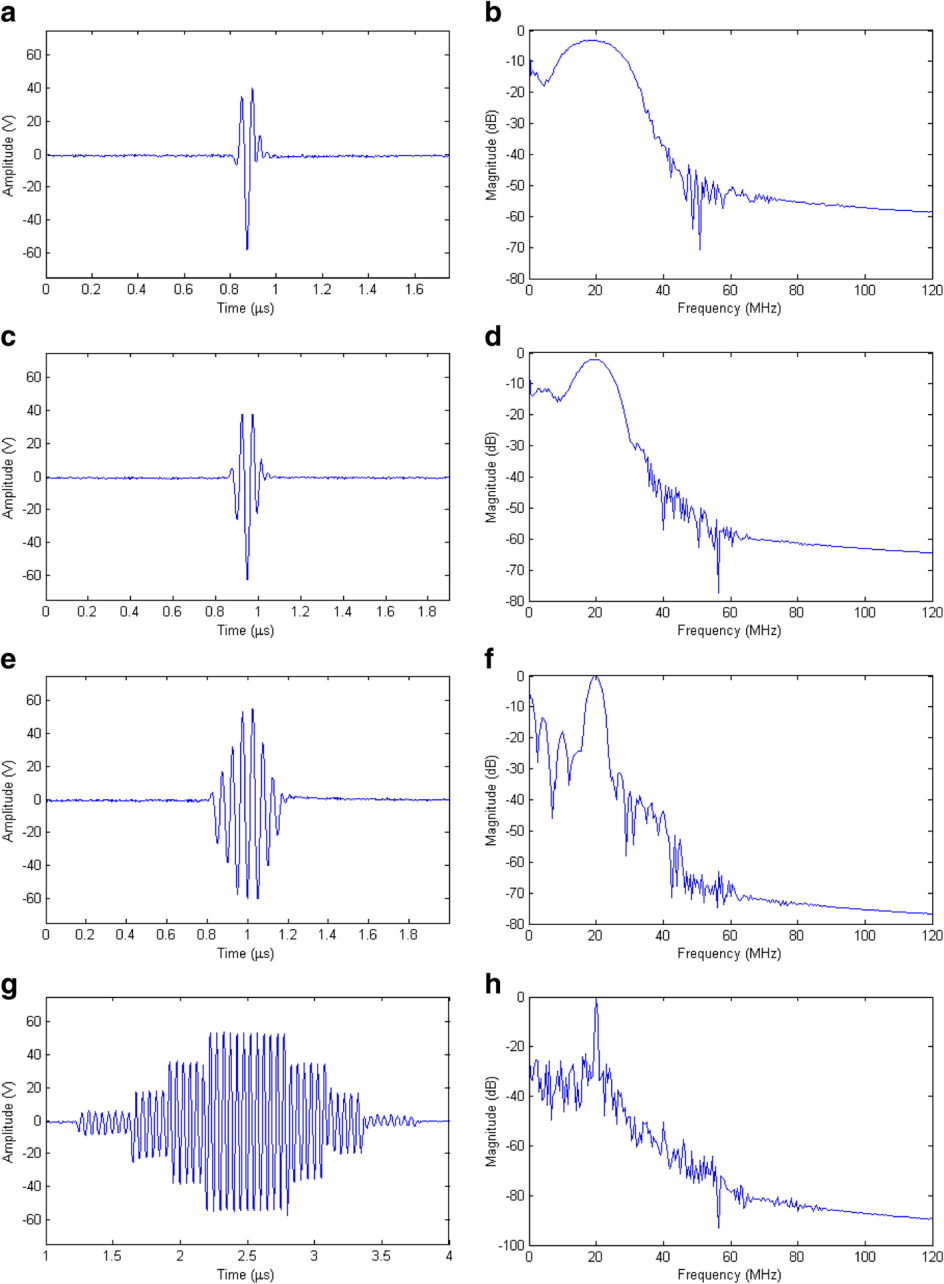
**High voltage 20 MHz pulses with the Gaussian profile measured on RC loads.** (**a**) A 1.5 cycle broad bandwidth pulse with 100 Vpp produced with −6 dB bandwidth (20.78 MHz) and (**b**) its spectrum with a relative bandwidth of 103.9%. (**c**) A medium bandwidth pulse and (**d**) its spectrum with a relative bandwidth of 54.2%. (**e**) A narrow bandwidth pattern and (**f**) its spectrum with a relative bandwidth of 23.1%. (**g**) A multicycle waveform for Doppler imaging and (**h**) its spectrum with a relative bandwidth of 3.6%.

As the pulse shape has a direct effect on axial resolution for resolving two adjacent objects separated along the acoustic axis [24], four different 20-MHz Gaussian-shaped pulses produced by the RAWG are shown in Figure 7. Figure 7(a) shows a 1.5 cycle pulse with amplitude of approximately 100 Vpp and a −6 dB bandwidth of 20.78 MHz, i. e., a relative bandwidth of 103.9% (Figure 7(b)). The pulse has a broad bandwidth radiation pattern suitable for detecting the size of small objects along the axis of the beam for B-mode imaging [19]. Figure 7(c) shows a medium bandwidth pulse appropriate for visualization of anatomical structures [17] with a relative bandwidth of 54.2% and Figure 7(d) its spectrum. A narrow bandwidth pattern with a relative bandwidth of 23.1% is shown in Figure 7(e) with its spectrum in Figure 7(f). This kind of pulse may be particularly useful in alternative methods for achieving dynamic transmit focus [1,27]. Figure 7(g) demonstrates that the RAWG can produce multicycle pulses up to 20 MHz with amplitude of 100 Vpp required for Doppler application [9,19] and Figure 7(h) its spectrum with a relative bandwidth of 3.6%. In all cases, the second harmonic of the produced pulses was less than −40 dB and the PRF can be adjusted for the requirements of imaging research.

**Figure 8:**
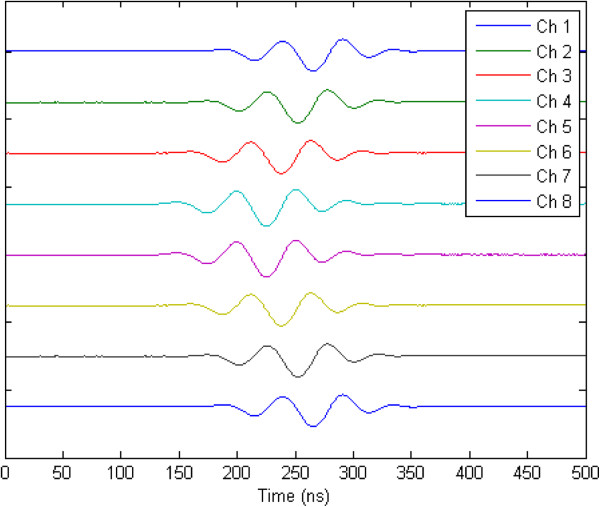
Experimental ultrasonic pulses emitted by the eight channels with same amplitude and phase angle control, and proper time delay for symmetrical focusing at 10 mm.

**Figure 9:**
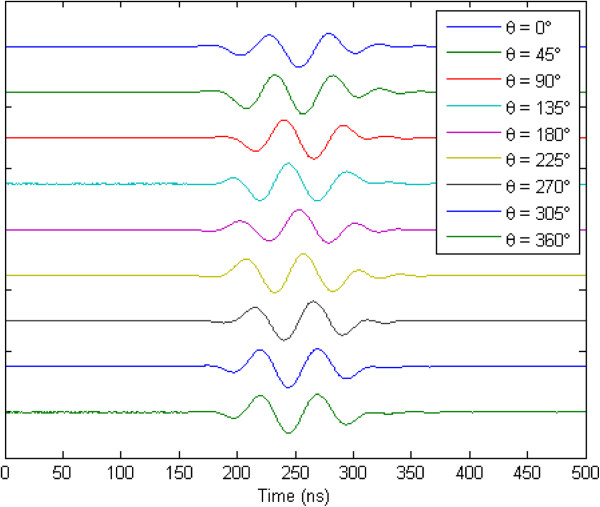
Comparison between the waveforms generated by one channel with phase angle from 0° to 360° with increment of 45°.

By controlling the excitation time, the resulting acoustic beam can be electronically focused onto different lines [22]. In order to give a quantitative example of the RAWG timing, considering that the speed of sound is 1540 m/s, the transducer parameters used to produce a focused beam pattern are summarized in Table 1. Based on these values, Figure 8 shows the experimental resulting eight waveforms generated with the same amplitude (*DAC* = 255) and phase angle control (0°), and individual time delay adjustment for focusing at 10 mm longitudinal waves with symmetrical delays about phase center. Additionally, fine focusing transmission phase adjustment can be performed and evaluated through the phase angle control. Figure 9 shows the comparison between the waveforms generated by one channel with phase angle from 0° to 360° with increment of 45°, as example for convenience.

**Table 1:** Transducer parameters used to produce a transmission focusing delay pattern

**Parameter**	**Value**
Number of elements	8
Center frequency (MHz)	20
Element pitch – kerf (mm)	0.008
Element height (mm)	1
Element width (mm)	0.3
Focal depth – FD (mm)	10

**Figure 10:**
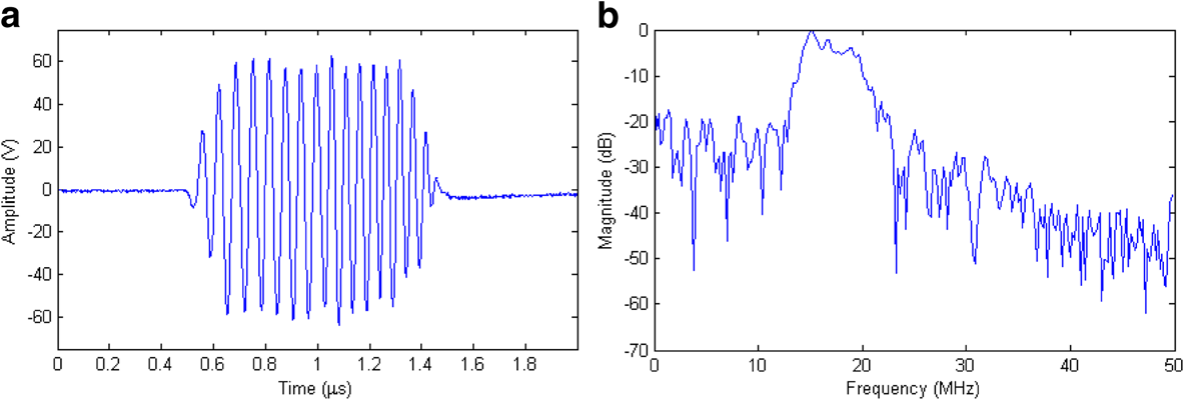
**Demonstration of a high voltage linear chirp-coded excitation.** (**a**) Chirp signal excitation between 15 and 20 MHz using a Tukey window with 12% taper ratio and duration of 1 μs and (**b**) its spectrum.

As an initial study to demonstrate the feasibility and validity of the proposed pulser, the RAWG was also programmed to generate a linear chirp-coded excitation, based on the work of Mamou *et al.*[28]. Figure 10(a) shows the result to produce a high voltage chirp signal between 15 and 20 MHz using a Tukey window with 12% taper ratio and duration of 1 μs, and Figure 10(b) its spectrum. According to Ricci *et al*. [10] and Qiu *et al*. [19], this initial result can be considered satisfactory for US imaging and the output waveform can be refined in future studies.

## Discussion

A high-frequency PWM modulation scheme was developed using four signals to control the necessary in-phase and quadrature look-up table timing to generate high voltage output waveforms with the Gaussian profile and adjustable amplitude.

The RAWG presented here generates complex excitation signals with a peak-to-peak voltage up to 120 Vpp at 10 MHz using a power supply of 70 V. Although such level is sufficient in most US applications, the proposed approach is able to operate with a high voltage supply up to 100 V. In this way, the choice to use the MD2130 beamforming source driver allowed us to overlap the limitation related to the electronics used for the amplification of the TX signals, described by Tortoli *et al*. [14], where the maximum output voltage level of the ULA-OP is fixed at 24 Vpp. Another important feature is the transmit time delay with resolution of 3.125 ns, which is adequate for high resolution transmitting waveform with appropriate focusing and side lobes reduction in the transmit beam. This parameter represents a potential limitation of the research platforms described in papers [4,14-16] to improve the performance in terms of quantization lobes. Also considering the TX section of such systems that uses high performance state-of-art FPGAs from Stratix family (Altera, San Jose, CA) and Virtex family (Xilinx, San Jose, CA) with a considerable cost, the proposed flexible transmission system was implemented using a Cyclone III FPGA Development Board (~US$ 1,200.00) with a relatively low cost FPGA (~US$ 500.00).

The achieved PWM clock frequency in this study (160 MHz) can be further improved using a new integrated pulser MD2131 (250 MHz) that was recently released by Supertex Inc. [29] to replace the MD2130 IC, featuring the same package and compatible pin-out configuration.

The parameters implemented in the FPGA can easily adjust beamforming settings through a GUI software to support different application requirements. Moreover, research users can also explore the parallel processing capability to implement alternative transmission strategies, reprogramming and reconfiguring the FPGA, and also adapting available Matlab, Visual C++ or others tools to develop a customized US research interface (URI) [5]. Different transmission sequences with time delay and phase adjustment can be transmitted to the MD2130 devices and arbitrarily changed between consecutive PRF through the individual 20 MHz SPI channel. Therefore, based on the initial result to produce a chirp signal (see Figure 10), we believe that the system programmability can meet the requirement for arbitrary waveform coded excitation with different windowing and modulated excitation imaging using various coding strategies, as described in papers [19,28,30].

Although this technique requires additional components compared to other US pulsers [8,9], and thus, considerably more area in a multichannel TX board, our preliminary experimental results show that the proposed research platform can be considerably advantageous to provide accurate control over several US transmission parameters, such as waveforms, aperture weighting amplitude control and dynamic focusing phase adjustment.

The proposed architecture was evaluated through onboard equivalent loads which includes a capacitor and resistor connected in parallel and performed exactly as expected, featuring low second order harmonic distortions (< −40 dB) and demonstrating its feasibility. On the other hand, this approach avoids the implementation of external DACs and broadband power amplifiers used in other research platforms [15,19] to generate the high-voltage pulses to properly drive the transducers. Thus, further research work is needed to demonstrate the feasibility of the RAWG with commercial transducers for different US applications and imaging modes. For example, we expect a close relationship between the amplitude of the excitation waveforms and the amplitude of echoes with low jitter and distortions, and also to evaluate the system performance as a high resolution transmit beamformer using wire phantoms and tissue mimicking phantoms with potential increase in SNR, which in turn will result in images with better resolution [24,25].

The breakdown voltage of the RAWG is up to 200 Vpp and the 3 A peak output current of the MD2130 push-pull source driver [26] ensures the driving capability on a capacitive load, which can result in significant signal loss due to transducer elements, connection cables and operating frequency [7,8]. At the same time, due to the nonidentical electrical characteristics between the passive components and some of the connecting traces lengths in the PCB layout, in particular between the MD2130 output pins and the two cascading DN2625 MOSFETs source pins, there was an output amplitude variation of 12 V and 13 V at 10 MHz and 20 MHz, respectively, across the RC loads. This difference can be minimized by refining the layout further as a future work.

More studies are required to optimize the presented system and facilitate its use on the research of new transmission investigation methods. In addition, the proposed hardware architecture can be further extended and developed to implement a complete US research system, including not only the TX but also the receive (RX) beamformer fully configurable and flexible, making it suitable for possible implementation of a large class of new US methods.

## Conclusions

In summary, we have successfully developed and tested a fully reconfigurable arbitrary waveform generator system specifically designed for US research purposes. The PWM technique has been efficiently implemented using LUTs in a low-cost FPGA, which controls eight MD2130 push-pull source drivers providing a suitable approach for generating simultaneous arbitrary waveforms over eight TX channels. The proposed RAWG system can be used in a wide range of US research applications, including novel TX beamforming methods, dynamic transmission focusing [27], high-intensity focused ultrasound (HIFU) [31], coded excitation [30] and others. The preliminary experimental results demonstrated the system flexibility to provide accurate beamforming and focus scanning for diagnostic and therapeutic US research applications, as well as non-destructive testing (NDT) image evaluation.

## Competing interests

The authors declare that they have no competing interests.

## Authors’ contributions

AAA participated in hardware design with JMM, carried out the study and prepared the manuscript. JMM proposed the idea, reviewed the results and written the manuscript. FKS corrected the manuscript and reviewed the results. VLSNB and ETC supervised the whole project and revised the manuscript. All authors read and approved the final manuscript.
